# Efficacy and Safety of Small Molecule Inhibitor Therapies for Vitiligo: A Systematic Review

**DOI:** 10.1002/hsr2.71842

**Published:** 2026-02-22

**Authors:** Alireza Jafarzadeh, Mohammad Amin Darvishi, Mina Khosravi, Masoumeh Roohaninasab, Azadeh Goodarzi

**Affiliations:** ^1^ Department of Dermatology Hazrat Fatemeh Hospital, School of Medicine Iran University of Medical Sciences Tehran Iran; ^2^ School of Medicine Iran University of Medical Sciences Tehran Iran

**Keywords:** apremilast, Janus kinase inhibitor, phosphodiesterase‐4 inhibitors, review, small molecule inhibitors, systematic review, tofacitinib, tyrosine kinase inhibitors, vitiligo

## Abstract

**Background and Aims:**

The latest advances in the treatment of vitiligo involve the introduction of Janus kinase inhibitors (JAKI) and small molecule inhibitors (SMI). These modern treatment modalities target specific inflammatory pathways that can improve outcomes for vitiligo in adults, adolescents, and children.

**Methods:**

We did a literature search among PubMed, Scopus, and Web of Science, utilizing PRISMA guidelines. Articles included in the study were those reporting systemic medications using JAKI and SMI for vitiligo, categorized into subgroups of children (2–12), adolescents (12–18), and adults (over 18). We extracted information based on patient demographics, treatment regimens, efficacy outcomes, adverse effects, and follow‐up data.

**Results:**

After screening 987 studies, a total of 25 articles met the inclusion criteria and were included. The analysis demonstrated that JAKI, as well as the phosphodiesterase‐4 Inhibitor (PDE4 Inhibitor), apremilast, showed notable efficacy in the treatment of vitiligo across various age groups. Among these, ritlecitinib was the most extensively studied, showing significant improvements in both Facial‐VASI and Total‐VASI scores, especially when combined with NB‐UVB phototherapy. Tofacitinib demonstrated up to 75% repigmentation, particularly in sun‐exposed areas and pediatric populations, with higher efficacy noted when used alongside phototherapy. Upadacitinib showed ≥ 35% improvement in Facial‐VASI scores, though higher doses were associated with increased adverse events, including a serious nonfatal ischemic stroke. Baricitinib led to > 50% VASI improvement in 70.6% of patients when combined with NB‐UVB. Apremilast showed partial disease control and up to 61.5% repigmentation, though it was generally less effective than corticosteroids in halting progression.

**Conclusions:**

JAKI and SMIs appear to be promising treatment options for vitiligo in adults, adolescents, and children, offering better efficacy than traditional treatments. However, some treatments like Apremilast had conflicting results about efficacy in vitiligo. Although these are notable findings, further research is required to establish their long‐term safety, particularly in children and adolescents.

**Abbreviations**
DLQI: dermatology life quality index
JAKI: Janus kinase inhibitor
NB‐UVB: narrowband ultraviolet B
NSV: nonsegmental vitiligo
PDE4: phosphodiesterase‐4
SMI: small molecule inhibitor
URI: upper respiratory infection
VASI: vitiligo area scoring index

## 1 Introduction

Vitiligo is an acquired, chronic depigmenting disorder characterized by the selective loss of melanocytes in the epidermis and, in some cases, skin appendages [1]. Although it is not a life‐threatening condition, vitiligo has a substantial psychosocial impact and is frequently associated with emotional distress, anxiety, and depression [2]. The global prevalence of vitiligo varies widely, ranging from approximately 0.06%–2.28% across different regions and populations [3]. In the United States, prevalence estimates range from 0.76% to 1.11%, affecting individuals of all skin types and ethnic backgrounds [4].

Vitiligo is widely considered an immune‐mediated disease. Current evidence indicates that cytotoxic autoreactive CD8⁺ T cells play a central role by inducing melanocyte apoptosis [5]. These T cells secrete pro‐inflammatory cytokines, particularly interferon‐*γ* (IFN‐*γ*), which contribute significantly to disease progression [6]. IFN‐*γ* signaling through the Janus kinase (JAK) pathway activates a self‐perpetuating inflammatory loop, leading to sustained T‐cell recruitment and persistence within affected skin [7].

Despite advances in understanding disease mechanisms, therapeutic options for vitiligo remain limited and often yield variable outcomes. Available treatments include narrowband ultraviolet B (NB‐UVB) phototherapy, topical and systemic immunosuppressive agents, and selected surgical approaches [8]. Systemic therapies, such as oral corticosteroids or immunomodulators, including methotrexate, may be used in rapidly progressive or extensive disease [9]. NB‐UVB phototherapy is considered the standard‐of‐care for generalized vitiligo; however, its efficacy is modest, with many patients achieving approximately 30% improvement in vitiligo area scoring index (VASI) scores [10].

Recent therapeutic developments have focused on JAK inhibitors, including tofacitinib, baricitinib, and upadacitinib, as potential treatments for vitiligo [11]. JAK inhibitors have demonstrated efficacy in several immune‐mediated dermatologic conditions, such as psoriasis, alopecia areata, and atopic dermatitis [12]. By inhibiting the JAK/STAT signaling pathway, these agents disrupt IFN‐*γ*‐mediated inflammation. For example, tofacitinib, a JAK1/3 inhibitor, has been shown to promote repigmentation by suppressing IFN‐*γ* signaling and reducing autoreactive T‐cell activity in the skin [13].

In addition to JAK inhibitors, other small‐molecule therapies have been investigated for vitiligo. Apremilast, an oral phosphodiesterase‐4 (PDE‐4) inhibitor, modulates immune responses by increasing intracellular cyclic adenosine monophosphate levels and reducing the production of pro‐inflammatory cytokines, including interleukin‐2, interleukin‐8, IFN‐*γ*, and tumor necrosis factor‐*α* [14]. Apremilast is currently approved by the U.S. Food and Drug Administration for the treatment of psoriatic arthritis, psoriasis, and oral ulcers associated with Behçet′s disease [15, 16, 17].

Although growing evidence supports the use of JAK inhibitors and other small‐molecule therapies in immune‐mediated diseases, data regarding their efficacy and safety in vitiligo remain limited, particularly across different age groups. Therefore, this study aims to systematically review available primary evidence—including randomized and nonrandomized clinical trials and case series—evaluating the use of JAK inhibitors and small‐molecule therapies in vitiligo. Additionally, we compare the reported efficacy and safety profiles of these agents among children, adolescents, and adults.

## 2 Methods and Materials

### 2.1 Search Strategy and Databases

**Figure 1:**
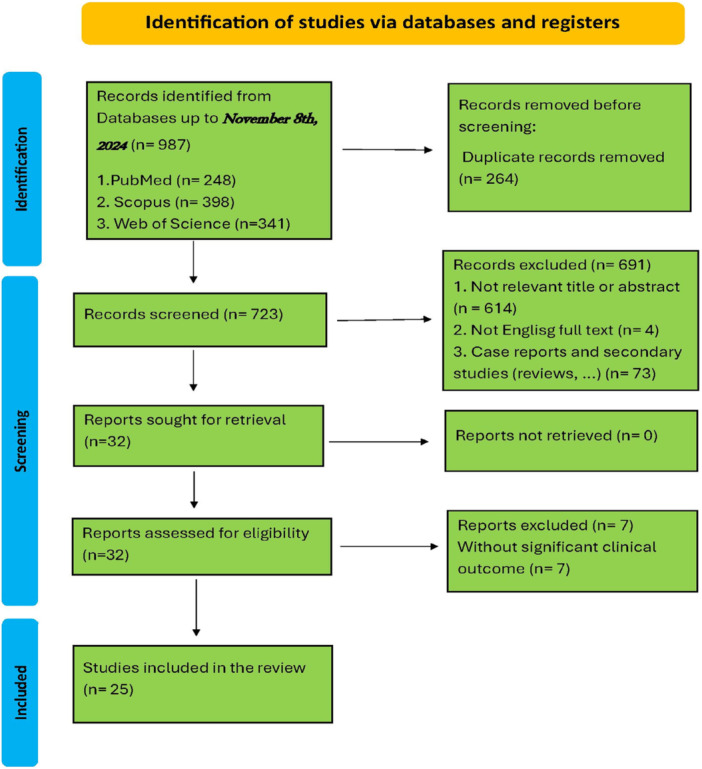
PRISMA 2020 flow diagram for new systematic reviews which included searches of databases and registers only.

This study was conducted in accordance with the guidelines established by the Preferred Reporting Items for Systematic Reviews and Meta‐Analyses (PRISMA) statement (Figure 1). A comprehensive literature search was performed to identify relevant studies investigating the use of JAK inhibitors and small‐molecule inhibitors (SMIs) in the treatment of vitiligo across different age groups, including adults, adolescents, and children (Table 1).

**Table 1:** Search syntax across databases.

Database	Search string	Number of results
PubMed	(“small molecule” OR “small molecule inhibitor” OR “Phosphodiesterase inhibitor” OR “PDE4 inhibitor” OR “Tyrosine kinase inhibitor” OR “TYK2 inhibitor” OR “apremilast” OR “roflumilast” OR “piclamilast” OR “Rolipram” OR “crisaborole” OR “orismilast” OR “cilomilast” OR “difamilast” OR “oglemilast” OR “tanimilast” OR “ibudilast” OR “lapatinib” OR “nintedanib” OR “osimertinib” OR “cobimetinib” OR “trametinib” OR “vemurafenib” OR “dabrafenib” OR “carfilzomib” OR “bortezomib” OR “romidepsin” OR “vorinostat” OR “sunifiram” OR “everolimus” OR “idelalisib” OR “fulvestrant” OR “axitinib” OR “dasatinib” OR “erlotinib” OR “imatinib” OR “tamoxifen” OR “tyrosine kinase inhibitor” OR “SERM” OR “SERD” OR “selective estrogen receptor modulator” OR “selective estrogen receptor degrader” OR “PI3k/Akt/mTOR pathway inhibitor” OR “Histone deacetylase inhibitor” OR “HDAC inhibitor” OR “proteasome inhibitor” OR “BRAF inhibitor” OR “MEK inhibitor” OR “ABBV‐181” OR “PF‐06750805” OR “JNJ‐64052981” OR “Lotamilast” OR “jak inhibitor” OR “Janus kinase inhibitor” OR “tofacitinib” OR “baricitinib” OR “ruxolitinib” OR “updacitinib” OR “fedratinib” OR “abrocitinib” OR “ritlecitinib” OR “deucravacitinib” OR “brepocitinib” OR “ropsacitinib” OR “filgotinib”) AND (“vitiligo”)	248
Scopus	TITLE‐ABS‐KEY (“small molecule” OR “small molecule inhibitor” OR “Phosphodiesterase inhibitor” OR “PDE4 inhibitor” OR “Tyrosine kinase inhibitor” OR “TYK2 inhibitor” OR “apremilast” OR “roflumilast” OR “piclamilast” OR “Rolipram” OR “crisaborole” OR “orismilast” OR “cilomilast” OR “difamilast” OR “oglemilast” OR “tanimilast” OR “ibudilast” OR “lapatinib” OR “nintedanib” OR “osimertinib” OR “cobimetinib” OR “trametinib” OR “vemurafenib” OR “dabrafenib” OR “carfilzomib” OR “bortezomib” OR “romidepsin” OR “vorinostat” OR “sunifiram” OR “everolimus” OR “idelalisib” OR “fulvestrant” OR “axitinib” OR “dasatinib” OR “erlotinib” OR “imatinib” OR “tamoxifen” OR “tyrosine kinase inhibitor” OR “SERM” OR “SERD” OR “selective estrogen receptor modulator” OR “selective estrogen receptor degrader” OR “PI3k/Akt/mTOR pathway inhibitor” OR “Histone deacetylase inhibitor” OR “HDAC inhibitor” OR “proteasome inhibitor” OR “BRAF inhibitor” OR “MEK inhibitor” OR "ABBV‐181" OR "PF‐06750805" OR "JNJ‐64052981" OR “Lotamilast” OR “jak inhibitor” OR “Janus kinase inhibitor” OR “tofacitinib” OR “baricitinib” OR “ruxolitinib” OR “updacitinib” OR “fedratinib” OR “abrocitinib” OR “ritlecitinib” OR “deucravacitinib” OR “brepocitinib” OR “ropsacitinib” OR “filgotinib") AND ("vitiligo")) AND (LIMIT‐TO (DOCTYPE,“ar"))	398
Web of Science	(“small molecule” OR “small molecule inhibitor” OR “Phosphodiesterase inhibitor” OR “PDE4 inhibitor” OR “Tyrosine kinase inhibitor” OR “TYK2 inhibitor” OR “apremilast” OR “roflumilast” OR “piclamilast” OR “Rolipram” OR “crisaborole” OR “orismilast” OR “cilomilast” OR “difamilast” OR “oglemilast” OR “tofimilast” OR “ibudilast” OR “lapatinib” OR “nintedanib” OR “osimertinib” OR “cobimetinib” OR “trametinib” OR “vemurafenib” OR “dabrafenib” OR “carfilzomib” OR “bortezomib” OR “romidepsin” OR “vorinostat” OR “sniiram” OR “everolimus” OR “idelalisib” OR “fulvestrant” OR “axitinib” OR “dasatinib” OR “erlotinib” OR “imatinib” OR “tamoxifen” OR “tyrosine kinase inhibitor” OR “SERM” OR “SERD” OR “selective estrogen receptor modulator” OR “selective estrogen receptor degrader” OR “PI3k/Akt/mTOR pathway inhibitor” OR “Histone deacetylase inhibitor” OR “HDAC inhibitor” OR “proteasome inhibitor” OR “BRAF inhibitor” OR “MEK inhibitor” OR “ABBV‐181” OR “PF‐06750805” OR “JNJ‐64052981” OR “Lotamilast” OR “jak inhibitor” OR “Janus kinase inhibitor” OR “tofacitinib” OR “baricitinib” OR “ruxolitinib” OR “upadacitinib” OR “fedratinib” OR “abrocitinib” OR “ritlecitinib” OR “deucravacitinib” OR “brepocitinib” OR “ropsacitinib” OR “filgotinib”) AND (“vitiligo”) (Topic) | 341 results	341
Total: 987.		
Total—all duplicates: 264.		
Total for screening: 723.		
Date of search: November 8, 2024.		

Systematic searches were carried out in the Scopus, Web of Science, and PubMed databases using predefined and database‐specific search strategies, as detailed in Table 1. The search encompassed all articles published up to November 8, 2024. To ensure completeness of the review, reference lists of all included studies were manually screened to identify any additional relevant publications that may not have been captured through the electronic database searches.

### 2.2 Inclusion and Exclusion Criteria

Studies were eligible for inclusion if they involved patients diagnosed with vitiligo aged older than 2 years, encompassing children, adolescents, and adults, irrespective of sex or ethnic background, and if they evaluated systemic therapeutic approaches using JAK inhibitors or other SMIs. Only primary research studies—including randomized controlled trials, nonrandomized clinical trials, cohort studies, and case series—that reported original and clearly distinguishable clinical outcomes related to vitiligo treatment were included in this review.

Studies were excluded if they failed to meet the aforementioned inclusion criteria. Specifically, case reports, secondary research articles such as narrative or systematic reviews, and studies focusing exclusively on topical therapies were not considered. In addition, investigations involving nonhuman subjects (including animal models or in vitro laboratory studies), studies lacking clinical outcome data or clearly defined outcome measures, and publications without accessible full‐text versions were excluded. Articles published in languages other than English or published prior to 2010 were also excluded from the analysis.

### 2.3 Study Selection and Data Extraction

Three viewers (M.D., A.J., and M.K.) assessed the titles and abstracts of the articles according to the qualification standards. The analysis of the articles contained different characteristics, like think about plan, the therapy approach, and the age run of members. One commentator (M.D.) extricated information from the last 24 articles. Member characteristics, counting cruel age, sexual orientation proportion, comorbidities, sort of illness, infection condition, and test measures, were recorded from the articles. Moreover, data on past medication sorts, ongoing medication use, treatment results and adequacy, unfavorable impacts, and security information were gathered. Rayyan.ai and Microsoft Word were utilized for screening the studies and extricating significant information. Three analysts conducted the screening, whereas one analyst was responsible for information extraction. Any contradictions were settled by meeting with a more expert analyst (A.J.).

### 2.4 Study Risk of Bias Assessment

**Figure 2:**
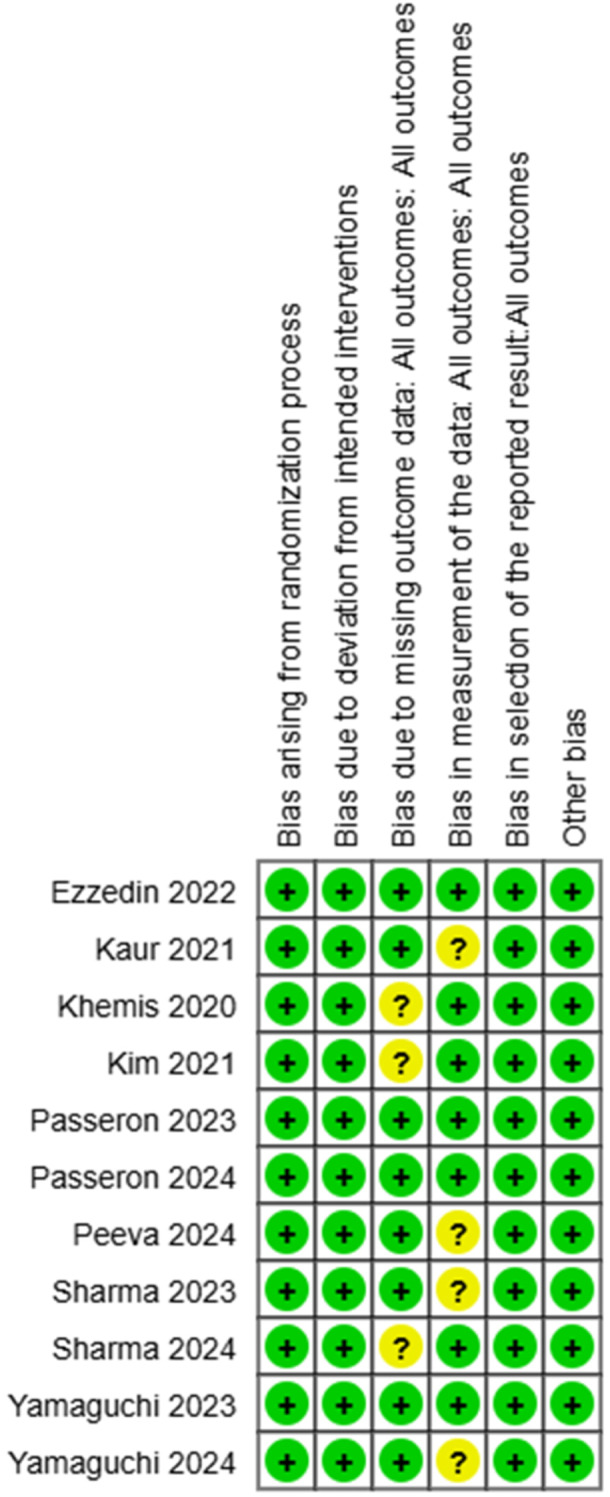
Quality assessment of the randomized articles involved in the review.

**Figure 3:**
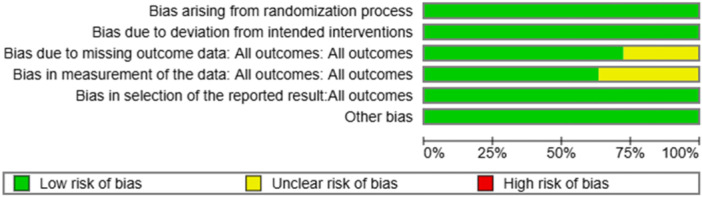
Assessment of the quality of the randomized articles involved in the review.

**Figure 4:**
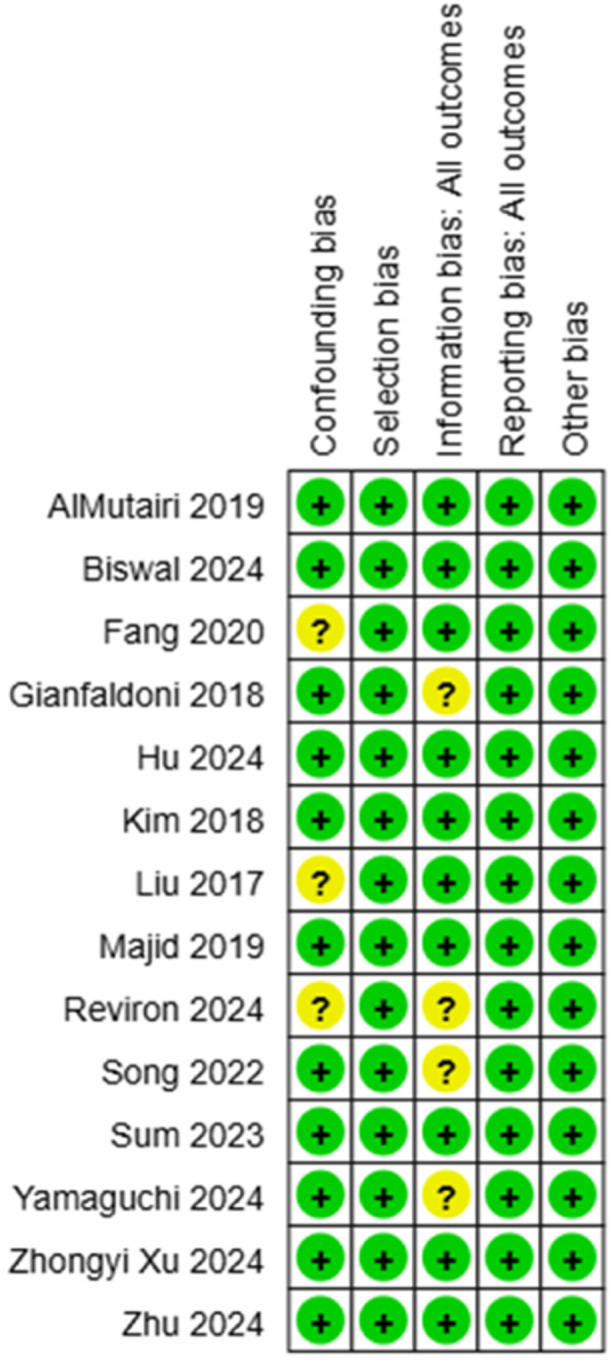
Quality assessment of the nonrandomized articles involved in the review.

**Figure 5:**
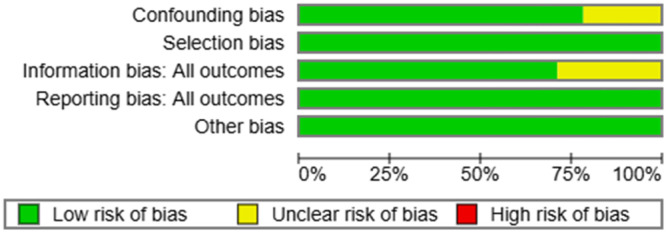
Assessment of the quality of the nonrandomized articles involved in the review.

One author (M.K.) surveyed the chosen articles utilizing the Cochrane Risk of Bias Tool for randomized Trials Version 2 (RoB2), together with the Exceed expectations instrument ROB2_IRPG_beta_v9, and for nonrandomized trials utilizing ROBINS‐1. This device incorporates an assessment of five domains, in addition to a general evaluation by the evaluator (Figures 2, 3, 4, 5).

### 2.5 Quality Assessment and Level of Evidence Evaluation

All included studies were stratified according to study design into randomized or controlled clinical trials, observational studies, and case series prior to data extraction. Given the heterogeneity of study designs and outcome measures, a formal quantitative risk‐of‐bias scoring was not undertaken. Instead, the methodological strength and level of evidence of each study were qualitatively assessed using established levels of evidence frameworks, with reference to the Oxford Centre for Evidence‐Based Medicine hierarchy. Randomized and controlled trials were considered higher‐level evidence, while observational studies and case series were classified as lower‐level evidence. This evidence stratification informed the synthesis and presentation of results, which were reported separately by study design and summarized in a high‐level evidence table.

### 2.6 Statistical Analysis

Statistical analyses were performed using SPSS Statistics version 28.0. Descriptive statistics were calculated for all baseline demographic variables, including age, gender, and disease duration. For between‐group comparisons, the *t*‐test was used for continuous variables, and the chi‐square test was employed for categorical variables. The significance level was set at *α* = 0.05 for all statistical tests.

A paired *t*‐test was used for comparing changes from baseline to follow‐up in VASI scores, with a two‐tailed significance level of *p* < 0.05. Effect sizes were calculated using Cohen′s *d* for significant findings to evaluate the magnitude of the effect. Exploratory analyses were conducted for subgroup analyses, such as patients under 18 years versus 18 years and older, to examine the influence of age on treatment efficacy.

All prespecified analyses were outlined in the study protocol and were conducted as planned. Post hoc analyses were performed to assess additional variables that were not prespecified, such as the impact of different treatment combinations on repigmentation. All tests were two‐sided unless stated otherwise.

### 2.7 Ethical Considerations and Study Registration

All collected data were kept confidential and analyzed without the inclusion of specific participant identifiers. The study adhered to the ethical principles outlined in the *Declaration of Helsinki*. The project was registered at *Iran University of Medical Sciences* under registration number *1403.470*. It was approved by the *Research Council* with the ethics code *IR.IUMS.FMD.REC.1403.470*. The study′s scientific title is “Efficacy and Safety of Small Molecule Inhibitor Therapies for Vitiligo: A Systematic Review in Adults, Adolescents, and Children.”

## 3 Results

### 3.1 Search Results

Through a systematic search process, a total of 987 potentially relevant records were identified, including 398 articles from Scopus, 341 from Web of Science, and 248 articles from PubMed. After the removal of duplicate records, 723 unique studies remained and were screened based on their titles and abstracts.

Subsequently, the full texts of 32 articles were independently assessed for eligibility by three reviewers (M.D., A.J., and M.K.). Following this evaluation, 25 studies met the predefined inclusion criteria and were selected for data extraction and qualitative synthesis. The study selection process is illustrated in detail in the PRISMA flow diagram presented in Figure 1.

### 3.2 Characteristics of Eligible Studies

A total of 25 studies including 2572 patients with vitiligo were included. By design, the evidence comprised 15 clinical trials, one observational study, and nine case series. To align with level‐of‐evidence principles, findings are presented stratified by study design: randomized/controlled trials (higher‐level evidence), observational studies (intermediate), and case series (lower‐level evidence).

Across all included studies, systemic therapies were distributed as follows: ritlecitinib (five studies; 1686 patients; 65.5%), apremilast (six studies; 369 patients; 14.4%), tofacitinib (eight studies; 180 patients; 7%), upadacitinib (two studies; 270 patients; 10.5%), baricitinib (two studies; 41 patients; 1.6%), and abrocitinib (one study; 11 patients; 0.4%). One study (4%) directly compared baricitinib, upadacitinib, and tofacitinib in 15 patients (0.6%) [18, 19, 20, 21, 22, 23, 24, 25, 26, 27, 28, 29, 30, 31, 32, 33, 34, 35, 36, 37, 38, 39, 40, 41, 42]. Detailed study and population characteristics are provided in Table 2.

**Table 2:** Characteristics of eligible studies.

Number	Study ID	Design of study	Sample size	Gender ratio (%F:M)	Age mean (range)	Age group	Past medical history and comorbidities	Type of disease	Disease condition(s)	Previous treatments	Treatment(s) of study	Outcome measurement and efficacy	Safety and adverse effects	Follow‐up evaluation
1	AlMutairi [25]	Prospective, open‐label, nonrandomized clinical trial	17 patients enrolled; 15 completed the study	41.2% female: 58.8% male (7F, 10M)	Not reported (21–58 years)	Adults	Unknown	Vitiligo	Progressive vitiligo which involves up to 10% of body surface area (BSA)	Topicals, systemic agents, phototherapy/lasers, and biologic agent discontinued for 2, 4, 8, and 12 weeks, respectively, before study	5 mg tofacitinib two times every day for 12 weeks	VASI score betterment appears with Face‐VASI scores seen in nine of 15 patients (75%). acral VASI betterment (up to 25%), seen in five of the 15 patients. The regimentation was kept up indeed after delaying the medication in all the patients.	No noticeable adverse effect was reported.	—
2	Yamaguchi et al. [20]	Prospective, randomized, double‐blind, placebo‐controlled, parallel‐group, multicenter, and dose‐ranging clinical‐trial Phase 2b	230 patients enrolled; 185 completed the study	57% female: 43% male (131F, 99M).	45 years (18–65 years)	Adults	Unknown	Nonsegmental vitiligo (NSV)	NSV and facial BSA involvement of > 0.25%	Any cell‐depleting agents, including rituximab, within 6 months; oral JAK inhibitors or biologics within 12 weeks; systemic treatments that could affect vitiligo, oral immune suppressants, intralesional steroid injection, or participation in other studies of investigational drugs within 8 weeks; phototherapy within 4 weeks; topical treatments that could affect vitiligo within 2 weeks; or herbal medications with unknown properties or known beneficial effects for vitiligo within 1 week	Within the dose‐ranging duration, participants were randomized to receive 50 mg ritlecitinib once daily with a 4‐week loading dose of 200 mg daily (200/50 mg), 50 mg ritlecitinib once daily with a 4‐week loading dose of 100 mg daily, 50 mg ritlecitinib once daily, 30 mg ritlecitinib once daily, 10 mg ritlecitinib once daily, or a placebo for 24 weeks. Patients concluding the 24‐week dose‐ranging phase were admitted to treatment during the expansion phase based on the response at Week 16 of the dose‐ranging phase. Patients exhibiting more than 50% change from baseline in the Add up to VASI (T‐VASI) score at Week 16 of the dose‐ranging phase received open‐label brepocitinib, open‐label ritlecitinib 200/50 mg alongside expanded NB‐UVB phototherapy twice weekly, or blinded ritlecitinib 200/50 mg.	At Week 24, the harsh change from baseline in the Facial‐VASI score was 57.0 vs. 51.5 (final perception carried forward; *p* = 0.15) and 69.6 vs. 55.1 (OC; *p* = 0.009), for ritlecitinib and NB‐UVB compared to ritlecitinib‐monotherapy, respectively. The harsh difference from the pattern in T‐VASI at Week 24 was 29.4 compared to 21.2 (final perception carried forward; *p* = 0.43) and 46.8 compared to 24.5 (OC; *p* < 0.001), respectively. The NB‐UVB extension to ritlecitinib was well tolerated with no unused safety signals.	UTI, URI, headache, nasopharyngitis, pruritus, and uterine leiomyoma in one patient, ritlecitinib monotherapy. Decreased absolute lymphocyte count, rise in LFT and CPK, rash, hearing loss, and photosensitivity	24‐week dose‐ranging period followed by a 24‐week extension period
3	Liu et al. [26]	Retrospective case series	10	50% female: 50% male (5F, 5M).	47.4 years (28–73 years)	Adults	Unknown	Vitiligo	Generalized vitiligo or primarily acral involvement, exhibiting 1%–100% BSA	NB‐UVB, topical tacrolimus, prednisone, topical steroids, pseudocatalase cream, blister grafting, secukinumab, excimer laser, topical and systemic PUVA, fraxel laser, and cryotherapy	5–10 mg tofacitinib QD‐BID for average 10 months	A cruel reduction of 5.4% BSA associated with vitiligo was observed in five out of 10 patients, while the remaining five patients did not achieve any noticeable repigmentation. Among the five patients who achieved some reversal of their condition, repigmentation occurred solely in sun‐exposed skin areas for 3 of them, diffusely in another patient undergoing simultaneous full‐body NB‐UVB phototherapy, and on the dorsal hands of yet another patient after starting concomitant hand NB‐UVB phototherapy.	URI, weight gain, arthralgias, and mild elevations of lipids	—
4	Kim et al. [27]	Case series	2	50% female 50% male (1F, 1M).	40 years (30–50 years)	Adults	Unknown	Vitiligo	Vitiligo with facial, neck, chest, and extreme lesions	Liquid nitrogen, topical corticosteroids, topical MBEH, and topical calcineurin. inhibitors, excimer laser, and NB‐UVB	The first one received 5 mg tofacitinib twice per day and NB‐UVB (400–500 mJ) twice per week, and the second one, 5 mg tofacitinib twice per day and NB‐UVB (360–500 mJ) two to three times per week, and only the face	The first patient received 5 mg tofacitinib two times daily and NB‐UVB (400–500 mJ) two times weekly, while the second patient was given 5 mg tofacitinib twice daily and NB‐UVB (360–500 mJ) two to three times weekly, focusing only on the face. After 3 months of treatment, the first patient′s face showed almost complete repigmentation. Seventy‐five percent or greater repigmentation of her neck, chest, forearms, and legs, with just minimal spotting on the dorsal hands. After 3 months, there was nearly 50% repigmentation of the face, and after 6 months, about 75% repigmentation was observed. No repigmentation occurred at the other areas of the body. No repigmentation occurred in other areas of the body.	No side effects reported	—
5	Ezzedine et al. [21]	Phase 2b, randomized, double‐blind, placebo‐controlled, parallel‐group, multicenter, and dose‐ranging clinical‐trial study	364 patients enrolled; 298 completed the study (one excluded due to Crohn′s disease).	53% female: 47% male (193F, 171M).	45 years (18–65 years)	Adults	Unknown	Vitiligo	BSA involvement of 4%–50% excluding palms, soles, and feet, BSA facial involvement $0.25%, excluding vermillions, and $1 active lesion, defined as new/extending lesion(s) in the past 3 months confirmed by photographs/medical record, confetti‐like lesion(s), trichrome lesion(s), or Koebner phenomenon/phenomena excluding history‐based isomorphic reaction	Any cell‐depleting agents, including rituximab, within 6 months; oral JAK inhibitors or biologics within 12 weeks; systemic treatments that could affect vitiligo, oral immune suppressants, intralesional steroid injection, or participation in other studies of investigational drugs within 8 weeks; phototherapy within 4 weeks; topical treatments that could affect vitiligo within 2 weeks; or herbal medications with unknown properties or known beneficial effects for vitiligo within 1 week	Two groups received a loading dose of 100 or 200 mg ritlecitinib day by day for 4 weeks, followed by support measurement of 50 mg day by day for 20 weeks; three groups without a loading dose of ritlecitinib received 50, 30, or 10 mg daily for 24 weeks, or using placebo for 24 weeks. Patients were accepted to treatment in the extension period as per the response at Week 16 of the dose‐ranging period. Nonresponders were accepted to an open‐label brepocitinib group, an open‐label ritlecitinib plus NB‐UVB therapy, or a blinded 200/50‐mg ritlecitinib group.	The results showed that significant differences from placebo in percent change from baseline in Facial‐VASI were observed for the ritlecitinib 50 mg groups with (21.2 vs. 2.1; *p* < 0.01) or without (18.5 vs. 2.1; *p* < 0.01) a loading dose and ritlecitinib 30 mg group (14.6 vs. 2.1; *p* = 0.01). Better results were observed after treatment with ritlecitinib 200/50 mg in the extension period.	Nasopharyngitis, URI, headache, nonserious herpes zoster, nonmelanoma skin cancers	A 24‐week dose‐ranging period was followed by a 24‐week extension period and follow‐up for 8 weeks.
6	Gianfaldoni et al. [28]	Observational retrospective study	67	65.6% female: 34.4% male (44F, 23M).	Not mentioned (25–61 years)	Adults	Arthritis rheumatoid for more than 3 years	Vitiligo Vulgaris	Steady or dynamic vitiligo duration between 2 and 10 years	10 mg tofacitinib per day	BIOSKIN EVOLUTION: a unique micro‐focused phototherapy cold light generator. Every 3 weeks, a single session is conducted for 12 sessions with an average dose of 50 mW/cm^2^. In the second group, 10 mg of tofacitinib is also administered daily.	42 patients who were treated with BIOSKIN achieved a repigmentation rate of more than 75%, with a mean rate of 77%. 11 patients fulfilled a significant betterment of the clinical results with a repigmentation rate between 50% and 75%; four patients experienced a moderate impact with a lesional repigmentation between 25% to 50%. Only one patient had an indigent impact to the phototherapy. All nine patients in combined therapy (tofacitinib plus BIOSKIN) fulfilled an almost full repigmentation of the vitiliginous lesions, with a repigmentation rate of 92%.	No side effect has reported.	—
7	Zhu et al. [36]	Prospective case series	15	53.3% female: 46.7% male (8F, 7M).	34.8 years (18–59 years)	Adults	Unknown	Vitiligo	Refractory vitiligo with Fitzpatrick skin Types III and IV	Intramuscular injection of betamethasone dipropionate monthly for 6 months	Five patients received tofacitinib 5 mg, five patients received baricitinib 2 mg, and five patients received padacitinib 15 mg monotherapy once daily over 6 months	The VASI score decreased form 10.39 ± 11.53 (tofacitinib), 12.12 ± 15.78 (baricitinib), and 13.02 ± 17.08 (padacitinib), at baseline to 5.81 ± 5.90, 8.15 ± 10.92, and 7.44 ± 9.73 at Month 6 (*p *= 0.146, *p*= 0.14, and *p*= 0.16, in order) The average VASI improvement reached 41.72 ± 8.42 (tofacitinib), 35.15 ± 10.12 (baricitinib), and 41.90 ± 8.87 (padacitinib) at Month 6 from 18.30 ± 7.15, 15.1 ± 8.55, and 16.73 ± 3.48 at Month 3 (*p*=0.005, *p*=0.006, and *p*=0.004, in order). The trichrome sign, confetti‐like sign, and Koebner phenomenon gradually disappeared across the three groups.	Three patients reported adverse effects. The most frequent was acne. No severe adverse effects were observed.	—
8	Xu et al. [35]	Prospective observational case‐series study	11	81.8% female, 18.2% male (9F, 2M)	35.9 years (30–59 years)	Adults	Unknown	Vitiligo	Refractory progressive vitiligo which persistent to treatment of six courses of intramuscular betamethasone injection	Six courses of intramuscular betamethasone injection	Oral abrocitinib 100 mg once daily for 16 weeks, and 10 of them (90.9%) decreased to 100 mg every other day for another 8 weeks.	The average BSA and VASI at the baseline were 10.3 ± 8.2 and 8.7 ± 6.8, which decreased to 9.7 ± 8.0 (*p* = 0.07) and 6.8 ± 5.0 (*p *= 0.04) at Week 24, indicating a mean improvement of 14.36% and 22.07%, respectively. The typical time from the dynamic phase to the steady phase was 2.0 ± 1.2 months. Six patients (54.5%) achieved a change of at least 25% in VASI, while two patients (18.2%) showed no improvement. In a group of seven patients with facial vitiligo, two patients (28.6%) achieved 75% improvement according to F‐VASI, two patients (28.6%) showed a 50% change in F‐VASI, one patient (14.3%) met the criteria for a 25% F‐VASI change, while two patients (28.6%) showed no improvement at all. Of the 10 patients with involvement of distal extremities, one patient (10%) achieved excellent (100%) repigmentation, two cases (20%), had moderate (50%–75%), one case (10%) showed mild (25%–50%), three cases (30%) exhibited minimal (1%–25%), and three cases (30%) did not show any change.	Three patients reported adverse effects, such as headache, dizziness, gastrointestinal discomfort, and nausea. Not any serious adverse effects were reported.	
9	Biswal et al. [18]	Case series	13	69.2% female, 30.8% male (9F, 4M)	11 years (6–16 years)	Children, adolescents	Unknown	Vitiligo	Progressive vitiligo resistant to topical treatment for more than 3 months, oral steroids, and cyclosporine for more than 6 weeks	Topical steroids and calcineurin inhibitors, PUVA, ayurvedic medications	5 mg tofacitinib twice per day orally with sun exposure for 5–10 min in 3–6 months	After 6 months, two patients exhibited nearly complete repigmentation, six patients demonstrated 70%–80% repigmentation, one patient was lost to follow‐up, and four patients did not respond to treatment. Areas exposed to the sun performed better than those that were covered. Overall, the vitiligo extent score (VES) indicated a significant improvement.	Grade 2 acne mildly deranged lipid profile	For any adverse effects or reactivation of the illness, patients were monitored for 6 months. One patient was not tracked after treatment.
10	Fang et al. [29]	A pilot case series study	4	50% female, 50% male (2F, 2M)	43 years (21–56 years)	Adults	Unknown	Vitiligo	Generalized vitiligo resistant to treatment, such as phototherapy at least 6 months	Phototherapy, at least for 6 months	5 mg daily tofacitinib, combined with NB‐UVB phototherapy	Three patients (2, 3, and 4) reported minor degrees of repigmentation, while Patient 4 noted a lack of improvement, with no change in the VES. The harsh reduction of VES for the other two patients showed a reaction of 24.9% (with a decrease of 46.3% in Patient 2 and 3.5% in Patient 3). Of the 111 vitiligo lesions, four patients exhibited notable cases, with 16 lesions showing varying levels of repigmentation (16/111, 14.4%). Patient 2 displayed the highest rate of lesions demonstrating signs of repigmentation (14/42, 33%). Two patients (3 and 4) each had one moderately repigmented lesion on their hand and trunk. The repigmentation pattern observed in most injuries resembled a nearly follicular design. The injury showed some signs of repigmentation after 16 weeks on the trunk (3/6, 50%), following injuries to the head and neck (7/16, 43.8%). Injuries to the hands, legs, and feet responded safely to the treatment regimen in this study. Due to Patient 2 exhibiting the optimal clinical response, an assistance investigation was conducted. The degree of repigmentation varied between districts in the ongoing 2. Three out of seven (42.9%) head and neck injuries and two out of four (50%) arm injuries showed significant improvement (refer to Review 3 and Review 4).	No patient had symptoms and signs of infection. No serious adverse events occurred during treatments.	—
11	Passeron et al. [31]	Phase II multicentre, randomized, double‐blind, placebo‐controlled, dose‐ranging clinical trial study	185	Unknown	46.5 years (18–65 years)	Adults	Unknown	Nonsegmental vitiligo (NSV)	Not mentioned	Unknown	Upadacitinib at different dosages of 6 mg (UPA6), 11 mg (UPA11), or 22 mg (UPA22), once a day for 24 weeks	Upadacitinib fulfilled the primary goal of change rate from baseline in F‐VASI at Week 24, with betterment in both UPA11 (−35.6%) and UPA22 (−34.0%) vs. placebo (−14.4%; *p* ≤ 0.01 and *p* ≤ 0.05), respectively. Patients satisfied more clinical response rates for F‐VASI with UPA11 (38.3% and 19.1%, individually) and UPA22 (39.5% and 14.0%, separately) vs. placebo treatment (10.9% and 2.2%, separately).	The unfavorable impacts were COVID‐19, skin breakout, cerebral pain, and nasopharyngitis. One serious adverse effect of nonfatal ischemic stroke was detailed.	After treatment for 24 weeks, a 28‐week follow‐up
12	Reviron et al. [33]	Retrospective case series	5	40% female, 60% male (2F, 3M)	60.2 years (39–79 years)	Adults	Psoriasis, Systemic lupus erythematosus	Vitiligo	Vitiligo which resistant to medication	Dermal corticosteroid, intravenous methylprednisolone, opical meladinine, methotrexate, oral methylprednisolone, tacrolimus ointment 0.1%, Heliotherapy, Psoralen.	4 mg baricitinib daily and direct characteristic heliotherapy (the consider characterized as 20 min of day by day sun introduction) for 5 months ± 1 month	The mean total BSA prior to starting therapy was 9.9% and shifted to 7.3% after 5 months (±1 month) of utilizing baricitinib. As a result, a harsh reduction of 25.9% (min: 10.4% and max: 41.9%) of the BSA was observed due to vitiligo in patients following 5 months (±1 month) of baricitinib in conjunction with heliotherapy. Patients with injuries in exposed areas like arms exhibited a strong response of repigmentation. The hands had areas of repigmentation that were very effective, although for the distal extremities.	Renal insufficiency (91ml/min to 67 ml/min in Month 4), CK elevation, TG elevation, LDL elevation	—
13	Passeron et al. [32]	Phase 2, multicentre, randomized, double‐blind, placebo‐controlled, dose‐ranging clinical trial study	185	62% female, 38% male (115F, 70M)	46.3 years (18–65 years)	Adults	Unknown	NSV	NSV with baseline scores of Facial VASI (F‐VASI) ≥ 0.5 and Total VASI (T‐VASI) ≥5.	Systemic vitiligo therapy (e.g., corticosteroids, methotrexate) or supplemental vitiligo therapy (e.g., antioxidants, herbal medicine) and any topical vitiligo therapy were discontinued at least 30 days before study. Phototherapy was discontinued for 12 weeks before the study. Sunlight exposure was allowed, but for prolonged exposure to sunlight, sunscreen was recommended.	Upadacitinib or Placebo 6 mg (UPA6), 11 mg (UPA11), or 22 mg (UPA22) once daily for 24 weeks	At Week 24, the LS mean difference compared to PBO in the percent change from baseline in F‐VASI was −7.60 (95% CI −22.18 to 6.97; *p* = 0.3) for UPA6, −21.27 (95% CI −36.02 to −6.52; *p* = 0.005) for UPA11, and −19.60 (95% CI −35.04 to −4.16; *p* = 0.01) for UPA22. The LS harsh contrast against PBO in the percent change from baseline in T‐VASI was −7.45 (95% CI −16.86 to 1.96; *p* = 0.1) for UPA6, −10.84 (95% CI −20.37 to −1.32; *p* = 0.02) for UPA11, and −14.27 (95% CI −24.24 to −4.30; *p* = 0.005) for UPA22. Ongoing treatment with upadacitinib led to sustained skin repigmentation over time without reaching a plateau by Week 52. The rates of study drug discontinuation and serious treatment‐emergent adverse events were greater in the UPA22 group compared to the UPA11 and UPA6 groups.	One death of unknown cause, one case of infiltrating lobular breast carcinoma, coronary artery arteriosclerosis, nonfatal ischemic stroke, COVID‐19, headache, acne, and fatigue	The study has 52 weeks of treatment plus 30 days of follow‐up after last dose of treatment.
14	Peeva et al. [22]	Phase 2b, randomized, double‐blind, placebo‐controlled, multicenter, dose‐ranging clinical trial	364 patients enrolled, 298 patients completed	53% female, 47% male (193F, 171M)	44.7 years (18–65 years)	Adults	Unknown.	NSV	NSV, at least one active lesion (new or extending lesion(s) within the last 3 months validated by images) or health history; resembling confetti lesion(s); trichrome lesion(s); or Koebner phenomenon/phenomena excluding Type 1 history‐driven isometric response, and influenced facial BSA of over 25%	—	Two teams were given an initial dose of ritlecitinib 100 or 200 mg each day for a duration of 4 weeks, subsequently using ritlecitinib 50 mg every day for 20 weeks. Three additional groups administered 50, 30, or 10 mg each day without an initial dose over a period of 24 weeks.	After 24 weeks, 50 mg of ritlecitinib enhanced the depigmentation assessed by percent change from baseline in facial‐VASI (placebo‐adjusted mean difference [90%CI]) for individuals with light (−15.2 [−24.7, −5.8]; *p* = 0.004) and dark (−37.4 [−50.3, −24.4]; *p* < 0.01) skin, showing continuous repigmentation up to Week 48. At Weeks 4 and 24, 50 mg of ritlecitinib reduced CXCL11 serum levels in patients with light skin, while those with dull skin experienced increased levels at Week 4 and no change at Week 24. Ritlecitinib 50 mg reduced IL‐9 and IL‐22 levels in dim skin in comparison to light skin.	Nasopharyngitis, URI, headache, herpes zoster, uterine leiomyoma	—
15	Sharma et al. [37]	Randomized pilot clinical trial	54 patients enrolled, 31 patients completed.	55.5% female, 44.5% male (30F, 24M)	31.6 years (not mentioned)	Adults	Unknown	NSV	Vitiligo Disease Activity (VIDA) score +4 and including more than 2% body surface.	2 weeks for topical medication and 1 month for phototherapy or systemic medication	30 mg apremilast twice every day or 2.5 mg betamethasone (oral mini‐pulse [OMP]) two times every week for 6 months.	The capture rate for vitiligo was found to be 60% with corticosteroid OMP after 6 months compared to 36% in the apremilast group. Although apremilast had significantly lower capture rates than OMP, it was linked to a rapid and marked reduction in the number of new vitiligo macules, although patients experienced a more variable illness progress.	Weakness, syncope, nausea, vomiting, diarrhea, gastroesophageal reflux, headache, appetite change, FBS elevation, high BP, leukocytosis	—
16	Sun et al. [19]	Retrospective case series	25	60% female, 40% male (15F, 10 M)	39.6 years (12–62 years)	Adolescents, adults	Unknown	NSV	NSV with a VIDA score of +4 and depigmented lesions involved 5%–50% of the body surface no improvement after 2–4 months of systemic corticosteroids	Systemic corticosteroids such as oral methylprednisolone, diprospan injection. topical corticosteroids and calcineurin inhibitors, and phototherapy	Tofacitinib 5 mg twice daily (2–9 months) and NB‐UVB phototherapy one to two times per week for 13–15 sessions as a concomitant treatment	After the medication, 16 of the 25 cases showed pausing illness progress, with VIDA score reduced to +3, +2, or +1. Period of illness control ranged between 1 and 4 months. Among them, seven patients progress was paused in 1 month, three patients in 2 months, four patients in 3 months, and two patients in 4 months. Nine patients had no clinical response to tofacitinib in this study. 10 of the 16 patients who fulfilled illness control had repigmentation in different ranges between 1 and 5 months. In two patients, clinical improvement was excellent (75% repigmentation), in three patients it was good (50%–75% repigmentation), and in three patients it was moderate (25%–50% repigmentation), in the other hand, two patients noted a poor clinical improvement (<25% repigmentation). Tofacitinib only controlled the illness progress, but without repigmentation in the other six cases. Finally, 10 patients with acceptable repigmentation, nine of them had attended NB‐UVB phototherapy. On the other hand, only one of the six patients without repigmentation had attended phototherapy.	Transient common cold	Follow‐up of 6 months, three patients continued to take low‐dose tofacitinib (5 mg every day), and 22 patients discontinued the treatment. Nine patients with no response to tofacitinib abandoned this therapy. Four patients remained in remission 6 months after discontinuation, and nine patients relapsed 2–5 months after discontinuation. Most of them received treatment with tofacitinib again when a relapse occurred.
17	Peeva et al. [23]	Phase 2b, randomized, dose‐ranging clinical‐trial study	364 patients enrolled, 253 completed	53% female, 47% male (193F, 171M)	44.7 years (18–65 years)	Adults	Unknown	NSV	NSV, ≥ 1 active lesion, and affected facial BSA of ≥0.25%	Unknown	Two groups received a loading dose of ritlecitinib 100 or 200 mg daily for 4 weeks, followed by ritlecitinib 50 mg daily for 20 weeks. Another three groups received 50, 30, or 10 mg daily without a loading dose for 24 weeks.	Regarding patients with lighter Fitzpatrick skin type (FST), the placebo‐related average change in F‐VASI at Week 24 for the 200/50, 100/50, 50, and 30 mg groups The mg groups were 14.3, 16.8, 14.6, and 10.4, respectively (*p *< 0.05 for all). group excluding 30 mg). For individuals with deeper FST, the placebo‐adjusted average %CFB in F‐VASI at Week 24 recorded values of 37.5, 37.3, 37.9, and 26.7, respectively (*p *< 0.05 for each team).	Nasopharyngitis, URI, headache, herpes zoster, uterine leiomyoma	In the extension, 187 patients were allocated to ritlecitinib 200/50 mg, and 136 (96 and 40 with lighter or darker FST, respectively) completed Week 48. Continuous repigmentation without stoppage of effect was seen in all FST through Week 48.
18	Song et al. [30]	Real‐world clinical practice	42 patients enrolled, 34 patients completed	26.5% female, 73.5 male (9F, 25M)	31 years (18–60 years)	Adults	No prior disease	Nonsegmental refractory vitiligo	Refractory vitiligo that had experienced limited improvement after at least 6 months of conventional therapies	Systemic treatments or phototherapy were discontinued 4 weeks before the study	In the first group, patients were treated with 5 mg tofacitinib two times every day orally, halometasone cream used for the lesions on the chest and limbs two times every day externally, and tacrolimus 0.1% ointment or pimecrolimus cream used for the face and neck twice a day externally. For combined therapy, NB‐UVB therapy was attended three times a week for a duration of 16 weeks. In the second group, patients were treated with halometasone cream, tacrolimus 0.1% ointment, or pimecrolimus cream as well as NB‐UVB therapy. The consumption of topical treatment and phototherapy in the second group was the same as that in the first group.	After 4 weeks, no noticeable change in the betterment of VASI was seen between the two groups (*p* > 0.05). However, from the eighth week, the repigmentation rate was notably more in the first group than the second one (*p* < 0.05). No noticeable changes in lesions on the face and neck were seen between the two groups after 16 weeks of treatment (*p* > 0.05). For acral lesions, however, the rate of repigmentation after 16 weeks was higher in the first group compared to the second one (*p* < 0.05). After 18 weeks, the repigmentation rate on the chest and limbs was notably more in the first group than the second one (*p* < 0.05).	Mild pain in thumb and hallux, burning pain or dryness of the skin and erythema after phototherapy, abnormal levels of blood lipids, uric acid, and coagulation function	—
19	Sharma et al. [38]	Prospective, open‐label, parallel‐group, randomized controlled trial	37 patients enrolled, 31 patients completed	43.2% female, 56.8 male (16F, 21M)	32.6 years (18–60 years)	Adults	Unknown	Unstable NSV	Unstable lesions are the lesions that have not been the same over 6 months and are continuously progressing or varying in shape and size.	Unknown	Oral apremilast 30 mg twice a day plus standard treatment for 12 weeks	The advance was seen in 93.75% of patients within the apremilast + standard gather vs. 66.66% of patients within the standard gather. However, on fisher′s exact test, the contrast was not noticeable between groups (*p* = 0.08). Repigmentation was noticed in 87.50% and 66.66% of the apremilast + standard and standard groups, respectively. The median duration to observe the first symptom of repigmentation was 1 month in the apremilast + standard group and 1.75 months in the standard group, supporting noticeably earlier repigmentation in apremilast + standard group (*p* = 0.01). After 12 weeks of study period, the median BSA score decreased noticeably (*p* = 0.02) in the apremilast + standard group from 12.50 (2.37–77.5) to 10.75 (4–73.7). However, the median BSA score in the standard group was the same (5.0) in both before and after treatment (*p *= 1.00). The median VASI score also decreased noticeably in the apremilast + standard group from 7.45 (0.9–39.9) to 6.21 (1.4–35.3) (*p *= 0.01); in the other hand, reduced in the standard group from 1.45 (0.4–5.8) to 1.4 (0.52–4.87) (*p *= 0.7). In both the groups, there was a noticeable reduction in dermatology life quality index (DLQI) score, from 4.40 ± 3.81 to 4.33 ± 3.33 (*p* = 0.02); also, in the apremilast + standard group, there was a noticeable (*p* = 0.003) contrast of 2.88 ± 3.66 in DLQI score from baseline, from 6.88 ± 5.03 to 4.00 ± 3.22 was seen. There is an increase of 8.67(±15.17) int he VASI in the standard group at 12 weeks, from 33.00 (±18.10) to 41.67 (±25.61) (*p *= 0.04). In the apremilast + standard group, there was a noticeable (*p *< 0.001) difference in VASI after 12 weeks, from 44.69 (±17.65) to 64.69 (±21.71), with an betterment of 20 (±16.63) in the group was a noticeable (*p*< 0.001) difference in VASI over 12 weeks, from 44.69 (±17.65) to 64.69 (±21.71), with an betterment of 20 (±16.63) in the group.	Weight loss, nausea, upper abdominal pain, appetite loss, insomnia, fatigue, URI, headache, and diarrhea. Depression in one patient	Each group was followed up after 2 weeks, 4 weeks, and then monthly after 2 and 3 months
20	Hu et al. [34]	Prospective, nonrandomized, controlled, open‐label, clinical‐trial study	36 patients enrolled, 33 patients completed	33.3% female, 66.7% male (12F, 24M)	34.2 years (18–60 years)	Adults	No prior disease	Progressive NSV	—	Systemic treatments or phototherapy were discontinued 4 weeks before the study	2 mg baricitinib every day in addition to NB‐UVB three times every week for 16 weeks	The study′s results confirmed that 12 of 17 (70.6%) patients in the combination group and two of 16 (12.5%) patients in the control group had a good VASI response (>50%) at Week 16 ([RR] = 5.6, 95% [CI] = 1.5–21.4, *p* < 0.001). At Week 16, a Facial‐VASI (> 75%) clinical answer was seen in 14 of 15 (93.3%) patients in the combination group, vs. five of 11 (45.5%) patients in the control group (RR = 2.1; 95% CI = 1.1–4.0; *p* = 0.02)	Erythema, slight blistering, and a burning sensation following phototherapy. Acne	—
21	Majid et al. [40]	Case series	13	38.5% female, 61.5% male (5F, 8M)	33.8 years (19–60 years)	Adults	Unknown	NSV	Rapidly progressive NSV involving 4%–30% BSA.	Systemic treatment or NB‐UVB was discontinued 3 months before the study	30 mg apremilast twice per day orally after titration of dosage for 3 months	Following a 3‐month titration of dosage, the treatment halted the progression of vitiligo in all cases, and no patient experienced an increase in their VASI score. Partial repigmentation occurred in eight out of 13 cases (61.5%) in various body areas such as the face, neck, trunk, and hands. Repigmentation was observed in regions where no topical treatment was applied. In addition, three out of the 13 cases exhibited vitiligo lesions on acral regions, and all of them noted either repigmentation or a reduction in the size of their lesions. The average reduction in VASI for the cases was 7.11% (95% CI 6.01–9.8), which was significant (*p* < 0.04). Every case met the treatment criteria, and the average satisfaction score for patients was 6.07 on a 1–10 scale.	Headache, nausea, vomiting, and abdominal discomfort	Check‐up biweekly for the initial month and subsequently each month thereafter. The main outcomes assessed at every follow‐up appointment were disease stabilization and repigmentation of wounds
22	Yamaguchi et al. [24]	Randomized, double‐blind, placebo‐controlled, Phase 2b clinical trial	364 patients enrolled; 298 completed the study (one excluded due to Crohn′s disease)	53% female: 47% male (193F, 171M).	45 years (18–65 years)	Adults	Unknown	NSV	NSV who had ≥1 active vitiligo lesion BSA of 4%–50% (excluding acral lesions) and facial BSA > 0.25% (excluding vermilions)	Any cell‐depleting agents, including rituximab, within 6 months; oral JAK inhibitors or biologics within 12 weeks; systemic treatments that could affect vitiligo, oral immune suppressants, intralesional steroid injection, or participation in other studies of investigational drugs within 8 weeks; phototherapy within 4 weeks; topical treatments that could affect vitiligo within 2 weeks; or herbal medications within 1 week	Daily ritlecitinib for 24 weeks, with or without a 4‐week loading dose: 200 mg (loading dose)/50 mg, 100/50 mg, 50 mg, 30 mg, 10 mg, or placebo	For all patients accepted in the Phase 2b trial, for active lesions, ritlecitinib effected in reductions of the progress of depigmentation in the 50‐mg group (+0.59 [–1.50, 2.68]; *p*= 0.009) and in the 30‐mg group (–1.45 [–5.47, 2.57]; *p*= 0.009) at Week 24 vs. placebo (+5.68 [2.59, 8.76]). A significant increase in depigmentation in the placebo was seen in active lesions. For stable lesions, ritlecitinib showed a significant reduction in depigmentation and an increase in repigmentation in the 50‐mg group (–6.35 [–8.45, −4.26]; *p* = 0.0016) and in the 30‐mg group (–7.98 [–12.95, − 3.01]; *p* = 0.009) at Week 24 compared with placebo (+0.51 [–2.89, 3.91]) Depigmentation rate did not differ in the placebo group in stable lesions.	Nasopharyngitis, URI, headache, nonserious herpes zoster, nonmelanoma skin cancers	A 24‐week dose‐ranging period was followed by a 24‐week extension period and an 8‐week follow‐up
23	Kaur et al. [39]	Prospective, randomized clinical trial	186	Unknown	Unknown	Adults	Unknown	NSV	Unknown	Unknown	Group 1 administered 30 mg apremilast daily; Group 2 administered 16 mg minipulse methylprednisolone orally; and Group 3 administered photochemotherapy.	Patients in all three groups reported betterment after the treatment duration. Group 2 detailed distant a much stronger response in unsteady vitiligo, taken after by Group 1 and after that by Group 3, respectively (*p*< 0.001).	Minor and transitory adverse effect	52 weeks of follow‐up every 4 weeks, measured by the VASI score and DLQI score
24	Khemis et al. [41]	Prospective randomized placebo‐controlled clinical trial study	79 patients enrolled, 72 patients	63.6% female, 36.3% male (49F, 28M)	47.4 years (unknown)	Adults	Unknown	NSV	Unknown	Unknown	Group A received, in addition to phototherapy, apremilast at the label dosage, and Group B received a placebo. After 24 weeks, patients who responded (decreased VASI > 30%) were rerandomized to receive apremilast or placebo, combined with twice weekly NB‐UVB for 24 additional weeks.	After 24 weeks, patients showing betterment (reduced VASI > 30%) were rerandomized to receive either apremilast or placebo, along with NB‐UVB twice a week for 24 weeks. At the end of 24 weeks of treatment, the median VASI score decreased from 12.90 (5.70–39.60) to 10.00 (5.50–24.30) (−4.12 ± 1.58; *p* = 0.01) in the apremilast + UVB group and from 13.90 (10.00–26.00) to 10.40 (5.10–20.00) (−6.81 ± 1.51; *p* < 0.01) in the placebo + UVB group. The difference between the two groups was not apparent (*p* = 0.18). Following 24 weeks of treatment, the median VES reduced from 9.05 (4.60–36.20) to 8.00 (4.00–25.00) (−3.38 ± 1.43; *p* = 0.02) in the apremilast + UVB cohort and from 10.00 (8.00–24.50) to 7.70 (5.20‐21.00) (−5.71 ± 1.37; *p* < 0.01) in the placebo + UVB cohort. The difference between the two groups was not significant (*p* = 0.2). Both groups experienced a slight yet clear decrease in their DLQI scores (*p* = 0.023 for apremilast + UVB and *p* = 0.004 for placebo + UVB). Once more, the difference between the two groups was not significant (*p* = 0.6). In the second phase, the median VASI score rose from 8.00 (4.70–28.10) to 9.00 (4.20–25.20) and (0.64 ± 1.15; *p* = 0.6) in the apremilast + UVB group, whereas it increased from 7.00 (5.10–14.40) to 7.50 (5.30–13.90) (−0.62 ± 1.15; *p* = 0.6) in the placebo + UVB group. The distinction between the two groups was not evident (*p* = 0.4).	Diarrhea, abdominal pain, and headache; two serious adverse events (one surgery for a benign tumor of the amygdala and one suicide attempt). The suicide attempt was attributed to the treatment.	The proportion of individuals in the apremilast + UVB group who lost their response during the second part of the study was three of 17 (17.7%) at Week 36, three of 15 (20%) at Week 48, and three of 12 (25%) at Week 52. In the placebo plus UVB group, six of 19 (31.6%) at Week 36, seven of 19 (36.8%) at Week 48, and four of 12 (33%) at Week 52 had lost their response. There was no statistical difference between the two groups at any of these time points.
25	Kim et al. [42]	Randomized, split‐body, pilot clinical‐trial study	28 subjects enrolled, 14 subjects completed	Unknown	Unknown	Adults	Unknown	Vitiligo	Not mentioned	Unknown	Regimen 1 included NB‐UVB phototherapy two or three times weekly during Stage 1 (Weeks 0–16), along with a half‐sided skin covering during Stage 2 (Weeks 16–32). Regimen 2 has been applied to the side that was secured during Stage 1, along with additional treatment using apremilast and NB‐UVB phototherapy in Stage 2 on the previously untreated side	The observed outcomes noted a higher chance of achieving satisfaction with 3 or 4 repigmentation sessions after 16 weeks of additional treatment using apremilast and NB‐UVB phototherapy compared to 16 weeks of NB‐UVB only (*p* = 0.001). The subsequent decrease in standard VASI score and BSA was observed after an additional 16 weeks of treatment compared to NB‐UVB alone (*p *= 0.01). No notable differences were observed in the DLQI and visual analog scale scores between the two groups (*p* = 0.05).	Adverse effects were known as side impacts of apremilast.	—

**Level‐of‐evidence tags (for transparency):** (Table 3).

RCTs = high/moderate (e.g., Oxford Level 1–2) Observational study = moderate/low (e.g., Oxford Level 2–3) Case series = low (e.g., Oxford Level 4).

**Table 3:** High‐level summary of evidence by study design.

Study design	Number of studies	Key interventions	Main outcomes assessed	Level of evidence	Overall strength of findings
Randomized/controlled clinical trials (RCTs)	15	Ritlecitinib, upadacitinib, baricitinib, apremilast, tofacitinib (adjunctive)	Facial‐VASI, Total‐VASI, disease stabilization, repigmentation rates, safety outcomes	High–Moderate	Consistent and statistically significant efficacy for ritlecitinib and upadacitinib; baricitinib effective as adjunct therapy; apremilast shows mixed but supportive results.
Observational study	1	Tofacitinib + phototherapy	Repigmentation rates, comparative response	Moderate–Low	Supports enhanced efficacy of tofacitinib when combined with phototherapy; limited by nonrandomized design.
Case series/case reports	9	Tofacitinib, baricitinib, abrocitinib, apremilast, comparative JAK inhibitors	Repigmentation patterns, disease stabilization, VASI/VES changes, adverse events	Low	Exploratory and hypothesis‐generating evidence; heterogeneous outcomes and limited generalizability

**(A) RCT (higher‐level evidence)**

**Adults (≥ 18 years)**

**A1. JAK Inhibitors**

**A1.1. Ritlecitinib—Five Clinical Trials (Higher‐Level Evidence)**

Five clinical trials evaluated oral ritlecitinib in 1686 adults aged 18–65 years.

**Trial 1 (dose‐ranging; 24 weeks; includes placebo):**

A total of 230 patients were randomized to ritlecitinib 50 mg QD after a 4‐week loading dose of 200 mg QD (200/50 mg) or 100 mg QD, ritlecitinib 50 mg QD without loading, 30 mg QD, 10 mg QD, or placebo. After the dose‐ranging phase, an extension phase was assigned by Week 16 response (e.g., those with >50% improvement in T‐VASI entered open‐label brepocitinib, open‐label ritlecitinib 200/50 mg + NB‐UVB twice weekly, or blinded ritlecitinib 200/50 mg). At Week 24, facial‐VASI improvement was greater with ritlecitinib + NB‐UVB versus ritlecitinib monotherapy (69.6 vs. 55.1; observed cases; *p* = 0.009, 95% CI: 4.5–7.5). T‐VASI improvements were also greater with combination therapy (46.8 vs. 24.5; *p* < 0.001). The addition of NB‐UVB was generally well tolerated without new safety signals. Reported adverse events included urinary tract infection, upper respiratory tract infection, headache, nasopharyngitis, pruritus, one case of uterine leiomyoma, transient reductions in absolute lymphocyte count, mild elevations in liver function tests and creatine phosphokinase, rash, hearing impairment, and photosensitivity, without a clear causal association [20].

**Trial 2 (randomized; 24 weeks; includes placebo):**

A total of 364 patients were randomized into five groups. Two groups received loading ritlecitinib (100 or 200 mg daily) for 4 weeks followed by 50 mg daily for 20 weeks. Three groups received 50, 30, or 10 mg daily for 24 weeks without loading; one group received placebo. Significant improvements in percent change from baseline in Facial‐VASI versus placebo were observed for ritlecitinib 50 mg with loading (21.2 vs. 2.1; *p* < 0.001) and without loading (18.5 vs. 2.1; *p* < 0.001) and for 30 mg (14.6 vs. 2.1; *p* = 0.01). Enhanced responses were noted in extension among the 200/50 mg regimen. Common adverse events included nasopharyngitis, upper respiratory infection, headache, nonserious herpes zoster, and nonmelanoma skin cancers [21].

**Trial 3 (randomized; 24 weeks; includes placebo; subgroup by skin type):**

In 364 patients, ritlecitinib 50 mg significantly improved facial depigmentation at Week 24 versus placebo in lighter skin types (−15.2; 90% CI: −24.7 to −5.8; *p* = 0.004) and darker skin types (−37.4; 90% CI: −50.3 to −24.4; *p* < **0.01**), with continued repigmentation through Week 48. Biomarkers: reductions in serum CXCL11 in lighter skin types at Weeks 4 and 24; transient increases at Week 4 with no significant change at Week 24 in darker skin types; reductions in IL‐9 and IL‐22 are more prominent in darker skin. Adverse events included nasopharyngitis, upper respiratory infection, headache, herpes zoster, and uterine leiomyoma [22].

**Trial 4 (randomized; 24 weeks; placebo‐adjusted facial‐VASI)**

Among lighter Fitzpatrick skin types, mean placebo‐adjusted improvements at Week 24 were 14.3 (200/50 mg), 16.8 (100/50 mg), 14.6 (50 mg), and 10.4 (30 mg) (*p* < 0.05 for all except 30 mg). For darker skin types, corresponding values were 37.5, 37.3, 37.9, and 26.7 (*p* < 0.05 for all groups). Safety profile remained consistent: nasopharyngitis, upper respiratory infection, headache, herpes zoster, and uterine leiomyoma [23].

**Trial 5 (Phase 2b; randomized; 24 weeks; dynamic vs. stable lesions)**

In 364 patients randomized to ritlecitinib 200, 100/50, 50, 30, 10 mg (±4‐week loading) or placebo, Week 24 showed significant reductions in depigmentation in both dynamic and stable lesions for 50 and 30 mg versus placebo. Dynamic lesions: 50 mg (0.59 [−1.50, 2.68]; *p* = 0.01) and 30 mg (−1.45 [−5.47, 2.57]; *p* = 0.009). Stable lesions: 50 mg (−6.35 [−8.45, −4.26]; *p* = 0.002) and 30 mg (−7.98 [−12.95, −3.01]; *p* = 0.009). Adverse events included nasopharyngitis, upper respiratory infection, headache, nonserious herpes zoster, and nonmelanoma skin cancers [24].

**A1.2. Tofacitinib—One randomized study + two non‐randomized/other trials (Higher‐to‐moderate evidence subset)**

Across adults, seven studies included two clinical trials, one observational study, and four case series (total 167 adults aged 18–73 years). Findings are separated by design below.

**Randomized study (42 patients; adjunctive topical therapy + NB‐UVB; 16 weeks)**

Two groups: (i) oral tofacitinib 5 mg twice daily + topical halometasone (trunk/extremities) and topical tacrolimus 0.1% or pimecrolimus (face/neck) + NB‐UVB three times weekly for 16 weeks; (ii) same topical therapies + NB‐UVB without tofacitinib. After Week 4, there was no difference in VASI improvement (*p* > 0.05). From Week 8 onward, repigmentation rates were significantly higher in the tofacitinib group (*p* < 0.05). No significant differences in face/neck at Week 16; acral lesions and trunk/extremities showed significantly greater repigmentation in the tofacitinib group at Weeks 16 and 18, respectively. Adverse events were mild: localized joint pain, burning/dryness, erythema after phototherapy, and transient laboratory abnormalities (lipids, uric acid, and coagulation) [30].

**Nonrandomized clinical trial (17 patients; 12 weeks)**

Oral tofacitinib 5 mg twice daily for 12 weeks. Improvement in VASI was mainly facial; facial VASI improvement in 9 out of 15 evaluable patients (75%). Acral improvement was limited to ≤ 25% in 5 out of 15. Repigmentation persisted after discontinuation in all responders. No clinically significant adverse events reported [25].

**A1.3. Upadacitinib—two randomized trials (higher‐level evidence)**

Two clinical trials in 270 adults aged 18–65 years.

**Trial 1 (randomized; 185 patients; 24 weeks)**

Upadacitinib 6 mg (UPA6), 11 mg (UPA11), 22 mg (UPA22) versus placebo. Primary endpoint met: mean percent change in F‐VASI at Week 24 in UPA11 (−35.6%) and UPA22 (−34.0%) versus placebo (−14.4%; *p* ≤ 0.01 and *p* ≤ 0.05). Higher proportions achieved clinically meaningful F‐VASI responses with UPA11 (38.3% and 19.1%) and UPA22 (39.5% and 14.0%) versus placebo (10.9% and 2.2%). Adverse events (AEs): COVID‐19 infection, skin rash, headache, and nasopharyngitis; one serious AE: nonfatal ischemic stroke [31].

**Trial 2 (randomized; 185 patients; 24 weeks; extension to Week 52)**

Least‐squares mean differences versus placebo in percent change in F‐VASI at Week 24: UPA6, −7.60 (95% CI: −22.18 to 6.97; *p* = 0.30); UPA11, −21.27 (95% CI: −36.02 to −6.52; *p* = 0.005); and UPA22, −19.60 (95% CI: −35.04 to −4.16; *p* = 0.01). T‐VASI differences versus placebo: UPA6, −7.45 (95% CI: −16.86 to 1.96; *p* = 0.10); UPA11, −10.84 (95% CI: −20.37 to −1.32; *p* = 0.02); and UPA22, −14.27 (95% CI: −24.24 to −4.30; *p* = 0.005). Sustained repigmentation occurred through Week 52 without plateau; higher discontinuation and serious treatment‐emergent AEs in UPA22 than in UPA11/UPA6. AEs included events of unclear etiology, invasive lobular breast carcinoma, coronary artery arteriosclerosis, nonfatal ischemic stroke, COVID‐19 infection, headache, acne, and fatigue [32].

**A1.4. Baricitinib—One randomized trial (higher‐level evidence component)**

**Randomized trial (36 patients; 16 weeks)**

Intervention, baricitinib 2 mg daily + NB‐UVB three times weekly for 16 weeks; control, NB‐UVB without baricitinib. At Week 16, good overall VASI response (> 50% improvement) in 12/17 (70.6%) versus 2/16 (12.5%) (RR = 5.6; 95% CI: 1.5–21.4; *p* < **0.001**). Facial‐VASI response > 75% in 14/15 (93.3%) versus 5/11 (45.5%) (RR = 2.1; 95% CI: 1.1–4.0; *p* = **0.02**). AEs were mild: erythema, minor blistering, burning after phototherapy, and acne [34].

**A2. Phosphodiesterase‐4 (PDE‐4) Inhibitor**

**A2.1. Apremilast—Five randomized trials (higher‐to‐moderate evidence; mixed consistency)**

Six studies total (five clinical trials + one case series) with 369 adults aged 18–60.

**RCT 1 (54 patients; 6 months):**

Apremilast 30 mg BID versus betamethasone oral mini‐pulse (OMP) 2.5 mg twice weekly. Disease stabilization: 60% (OMP) versus 36% (apremilast). OMP superior for disease control; apremilast associated with rapid reduction in new lesion development. Neither produced substantial repigmentation; QoL outcomes emphasized. Overall: apremilast less effective than OMP for halting progression but may slow disease activity [37].

**RCT 2 (37 patients; 12 weeks):**

Apremilast 30 mg BID + standard therapy versus standard therapy alone. Clinical improvement: 93.8% versus 66.7% (*p* = 0.08). Repigmentation: 87.5% versus 66.7%. Median time to initial repigmentation shorter with apremilast (1 vs. 1.75 months; ***p* = 0.018**). Significant reductions in median BSA and VASI only in apremilast + standard group. DLQI improved in both groups, greater reduction with apremilast. AEs: weight loss, nausea, abdominal pain, appetite loss, insomnia, fatigue, URIs, headache, diarrhea, one case of depression [38].

**RCT 3 (186 patients; three arms):**

Apremilast 30 mg daily versus oral minipulse methylprednisolone versus photochemotherapy. Improvement across all arms; minipulse corticosteroid most pronounced in unstable vitiligo, followed by apremilast then photochemotherapy (*p* < 0.001). Apremilast AEs mild/transient [39].

**RCT 4 (79 patients; 24 weeks + extension):**

Apremilast + NB‐UVB versus placebo + NB‐UVB. Both groups improved (VASI, VES, and DLQI), but no significant between‐group differences; extension did not yield further significant improvements. AEs: GI symptoms, headache. Two serious AEs: surgery for benign tumor and one suicide attempt (possibly related to apremilast) [41].

**RCT 5 (28 patients; two‐stage protocol; 16 weeks):**

NB‐UVB alone versus apremilast + NB‐UVB. Combination achieved higher rates of moderate‐to‐marked repigmentation after 16 weeks (*p* = 0.001), with additional reductions in VASI and BSA involvement (*p* = 0.01). No significant differences in DLQI or VAS; AEs consistent with known apremilast profile [42].

**B) Observational study (intermediate evidence)**

**B1. Tofacitinib (observational component; 67 patients)**

Patients were divided into two groups: BIOSKIN EVOLUTION microfocused phototherapy every 3 weeks for 12 sessions (mean dose 50 mW/cm²) versus the same phototherapy plus oral tofacitinib 10 mg daily. Phototherapy alone: 42 patients achieved >75% repigmentation (mean 77%), 11 achieved 50%–75%, 4 achieved 25%–50%, and 1 minimal response. Combination therapy: all nine patients achieved near‐complete repigmentation, mean 92%. No treatment‐related adverse events were reported [28].

**C) Case series/Case reports (lower‐level evidence)**

**C1. Children (2–12 years)—Tofacitinib case series (low evidence)**

A pediatric case series investigated tofacitinib for progressive vitiligo in patients aged 6–16 years (13 patients). Oral tofacitinib 5 mg BID plus controlled sun exposure (5–10 min) for 3–6 months. At 6 months: near‐complete repigmentation in two patients; six patients achieved ~70%–80% repigmentation; four had no significant response; one lost to follow‐up. Repigmentation was more pronounced in sun‐exposed areas; clinically meaningful improvement in Vitiligo Extent Score (VES) was reported. AEs: grade 2 acne and mild lipid elevations [18].

**C2. Adolescents (12–18 years)—Tofacitinib case series (low evidence)**

Two case series evaluated tofacitinib in progressive vitiligo and NSV.

**Case series 1 (same pediatric series; statistical reporting included):**

Paired *t‐*test showed significant improvement (*p* = 0.03; 95% CI: 0.10–0.50) with moderate effect size (Cohen's *d* = 0.45) [18].

**Case series 2 (25 patients; 2–9 months + NB‐UVB):**

Tofacitinib 5 mg BID for 2–9 months + NB‐UVB once/twice weekly (13–15 sessions). Disease stabilization in 16 patients (64%) with VIDA score reduction from +4 to +3/+2/+1. Time to stabilization: 1–4 months (7 within 1 month; 3 within 2; 4 within 3; 2 within 4). Nine did not respond. Repigmentation in 10 patients within 1–5 months: excellent (≥ 75%) 2; good (50%–75%) 3; moderate (25%–50%) 3; poor (<;25%) 2. Six additional patients stabilized without repigmentation. Nine of 10 repigmenters had adjunctive NB‐UVB. No serious AEs; minor AE: transient URI resembling common cold (19). Overall: 64% stabilization, moderate effect size (Cohen's *d* = 0.53), statistically significant (*p* = 0.04; 95% CI: 0.12–0.60).

**C3. Adults—Tofacitinib case series (low evidence; heterogeneous)**

**Case series (10 patients; 9.9 months mean):**

Tofacitinib 5–10 mg once/twice daily. Mean reduction 5.4% BSA involvement in five patients; five had no repigmentation. Responders: sun‐exposed areas (3), diffuse with full‐body NB‐UVB (1), dorsal hands after localized NB‐UVB (1). AEs: URIs (2), mild weight gain (1), arthralgia (1), mild lipid elevations (4) [26].

**Two‐patient series (tofacitinib 5 mg BID + NB‐UVB):**

NB‐UVB 400–500 mJ twice weekly (patient 1) and 360–500 mJ two–three times weekly to face only (patient 2). At 3 months: patient 1 near‐complete facial and >75% neck/chest/forearms/legs with minimal dorsal hands response; patient 2 ~50% facial at 3 months progressing to ~75% at 6 months with no repigmentation elsewhere. No AEs reported [27].

**Case series (four patients; tofacitinib 5 mg daily + NB‐UVB):**

Three patients improved; one no VES improvement. Responders had mean VES reduction 24.9% with variability. Of 111 lesions, 16 (14.4%) repigmented (mostly follicular). Trunk/head‐neck responded better; acral lesions largely resistant. No infections/serious AEs [29].

**C4. Baricitinib case series (low evidence)**

Five adults received baricitinib 4 mg daily + heliotherapy (~20 min/day) mean 5 months (±1). Mean BSA decreased 9.9%–7.3% (mean relative reduction 25.9%; range 10.4%–41.9%). Better response in sun‐exposed areas; hands improved; distal extremities less robust. AEs: eGFR decline (91 → 67 mL/min by month 4), elevations in CK, triglycerides, LDL [33].

**C5. Abrocitinib case series (low evidence)**

Eleven adults (30–59) received abrocitinib 100 mg daily for 16 weeks then 100 mg every other day for 8 weeks. Baseline mean BSA 10.3 ± 8.2; VASI 8.7 ± 6.8. By Week 24: BSA 9.7 ± 8.0 (*p* = 0.078), VASI 6.8 ± 5.0 (*p* = 0.039), mean improvements 14.36% and 22.07%. Time to transition from active to stable phase: 2.0 ± 1.2 months. Six patients (54.5%) achieved ≥25% VASI improvement; two (18.2%) no measurable improvement. Facial involvement (*n* = 7): ≥75% F‐VASI (2; 28.6%), ~50% (2; 28.6%), 25% (1; 14.3%), no response (2; 28.6%). Distal extremities (*n* = 10): complete (1; 10%), 50%–75% (2; 20%), 25%–50% (1; 10%), 1%–25% (3; 30%), no change (3; 30%). AEs mild in three patients (headache, dizziness, GI discomfort, and nausea); no serious AEs [35].

**C6. Apremilast case series (low evidence)**

One case series evaluated apremilast in 13 adult patients treated with oral apremilast 30 mg twice daily for 3 months. Complete arrest of disease progression was achieved in all patients. Partial repigmentation was observed in eight patients (61.5%), involving the face, neck, trunk, hands, and acral areas. The mean reduction in Vitiligo Area Scoring Index (VASI) was 7.11% (95% CI: 6.01–9.80; *p* < 0.04). Patient‐reported satisfaction was moderate to high, with a mean score of 6.07 on a 10‐point scale. Reported adverse events were mild and included headache, nausea, vomiting, and abdominal discomfort (40).

**C7. Comparative case series (low evidence): Baricitinib versus upadacitinib versus tofacitinib**

Fifteen adults (three groups of five) received: tofacitinib 5 mg daily, baricitinib 2 mg daily, upadacitinib 15 mg daily for 6 months. Mean VASI decreased from 10.39 ± 11.53 to 5.81 ± 5.90 (tofacitinib), 12.12 ± 15.78 to 8.15 ± 10.92 (baricitinib), 13.02 ± 17.08 to 7.44 ± 9.73 (upadacitinib) at Month 6; not statistically significant (*p* = 0.146; 0.10; 0.16). However, mean percentage VASI improvements increased significantly from Month 3 to Month 6: 18.30 ± 7.15 → 41.72 ± 8.42 (*p* = 0.005); 15.17 ± 8.55 → 35.15 ± 10.12 (*p* = 0.006); 16.73 ± 3.48 → 41.90 ± 8.87 (*p* = 0.004). Disease activity features (trichrome sign, confetti‐like depigmentation, Koebner phenomenon) gradually resolved. No major AEs; mild AEs in three patients, acne most frequent [36].

#### 3.2.1 Safety

Overall, the reviewed studies reported no unexpected safety signals, and the majority of adverse events were mild to moderate in severity and self‐limiting in nature. Nevertheless, several serious adverse events were documented and appeared to be drug‐specific.

Abrocitinib was most commonly associated with neurological and gastrointestinal adverse effects, including headache, dizziness, nausea, and gastrointestinal discomfort. In studies evaluating ritlecitinib, reported serious adverse events included hearing impairment, photosensitivity reactions, herpes zoster infection, nonmelanoma skin cancers, and isolated cases of uterine leiomyoma. Baricitinib therapy was associated with renal function impairment and related laboratory abnormalities, including elevations in creatine kinase, low‐density lipoprotein cholesterol, and triglyceride levels.

Upadacitinib demonstrated a more concerning safety profile in some studies, with reports of serious adverse events such as invasive lobular breast carcinoma, coronary artery arteriosclerosis, nonfatal ischemic stroke, and one death of unknown etiology. Apremilast was associated with a range of systemic adverse effects, including syncope, hypertension, insomnia, weight loss, and depressive symptoms. Notably, two serious adverse events were reported in one apremilast study: surgical intervention for a benign tumor and a suicide attempt, the latter considered potentially related to treatment.

## 4 Discussion

This systematic review synthesized evidence from 25 studies encompassing a total of 2572 patients, evaluating the efficacy and safety of JAK inhibitors and SMIs in the treatment of vitiligo across different age groups, including children, adolescents, and adults.

In pediatric populations, available evidence suggests that JAK inhibitors—particularly tofacitinib—may provide meaningful therapeutic benefits by promoting repigmentation of depigmented lesions [18]. These findings support the potential role of JAK inhibition in childhood vitiligo. Notably, no primary studies were identified that specifically evaluated the efficacy of other SMIs in pediatric patients, highlighting an important gap in the literature.

Among adolescents, JAK inhibitors—again most notably tofacitinib—demonstrated favorable clinical outcomes, with significant repigmentation reported across available studies [18, 19]. Similar to pediatric populations, no primary studies assessing the efficacy of non‐JAK SMIs in adolescents were identified. Collectively, these findings further support JAK inhibitors as promising therapeutic agents for vitiligo in adolescent patients.

In adult populations, a substantial body of evidence supports the efficacy of JAK inhibitors for vitiligo treatment. Ritlecitinib consistently demonstrated clinically meaningful effects in halting disease progression, with several studies reporting sustained benefits even after treatment discontinuation [20, 21, 22, 23, 24]. Tofacitinib showed efficacy across multiple studies, either as monotherapy or in combination with phototherapy [19, 25, 26, 27, 28, 29, 30]. Upadacitinib yielded significant improvements in vitiligo outcomes across different dosing regimens [31, 32], while baricitinib—evaluated in two studies—demonstrated promising results as an emerging JAK inhibitor in adult patients [33, 34]. Evidence regarding abrocitinib remains limited, with only a single case series reporting modest clinical improvement [35]. Additionally, one comparative case series suggested that baricitinib, upadacitinib, and tofacitinib all exhibited therapeutic potential at varying dosages [36]. Overall, these findings reinforce the efficacy of JAK inhibitors in adult vitiligo management.

With respect to other SMIs, apremilast—a PDE‐4 inhibitor—was the only agent evaluated across multiple studies. However, the results regarding its efficacy were inconsistent. Several trials failed to demonstrate superiority over standard therapies such as narrowband ultraviolet B (NB‐UVB) phototherapy or corticosteroids [37, 39, 41]. In contrast, other studies reported beneficial clinical effects of apremilast in adult patients [38, 40, 42]. Given these conflicting findings, definitive conclusions regarding the effectiveness of apremilast in vitiligo cannot currently be drawn. Overall, JAK inhibitors appear to provide more consistent therapeutic benefits compared with PDE‐4 inhibitors such as apremilast, although direct comparative studies remain limited.

Regarding safety, most adverse events reported across the included studies were mild to moderate in severity; however, serious adverse events were documented with certain JAK inhibitors, particularly ritlecitinib and upadacitinib [20, 21, 22, 23, 24, 31, 32]. Apremilast was also associated with serious adverse outcomes in one study, including a suicide attempt and surgical intervention for a benign tumor [41]. These findings underscore the importance of careful patient selection and vigilant long‐term safety monitoring.

Previous systematic reviews in this field have predominantly focused on adult populations and often excluded pediatric and adolescent patients [43, 44, 45, 46]. Moreover, long‐term safety data remain limited. Although PRISMA guidelines were followed, restricting the review to English‐language publications and three major databases may have resulted in the omission of relevant studies.

The findings of this review have important implications for clinical practice and future research. Treatment strategies should be individualized according to patient age, disease characteristics, and risk–benefit considerations, with close monitoring of growth and developmental milestones in younger populations. Standardized outcome measures, cost considerations, equitable access to emerging therapies, and structured long‐term safety surveillance are essential. Future studies should prioritize long‐term safety and include diverse populations across different geographic regions.

## 5 Limitations

One limitation of this review was the focus on primary studies, including clinical trials and case series, which led to the exclusion of data from secondary studies and case reports that have explored the efficacy of JAK inhibitors and SMIs in the treatment of vitiligo across different age groups. Additionally, limited access to the full text of certain publications restricted the inclusion of some potentially relevant studies.

## 6 Conclusion

In this review, we evaluated the efficacy of JAK inhibitors and SMIs as therapeutic options for vitiligo across pediatric, adolescent, and adult populations, drawing on evidence from primary studies including clinical trials and case series. Overall, the findings suggest that these agents may be effective in disease control and, in some cases, in promoting repigmentation. However, the evidence regarding certain therapies—particularly apremilast—remains inconsistent, with conflicting results reported across studies.

These observations indicate a potential shift away from conventional treatment modalities, which are often associated with adverse effects that may limit their long‐term use. JAK inhibitors and selected SMIs may represent promising alternatives, given their targeted mechanisms of action, potentially improved disease control, and more favorable tolerability profiles compared with standard therapies.

Future research should focus on well‐designed, large‐scale studies to further evaluate the efficacy and long‐term safety of these agents across different age groups, particularly in children and adolescents, where evidence remains limited. Such studies are essential to establish age‐specific treatment guidelines and to clarify the optimal role of these therapies in the comprehensive management of vitiligo.

## Author Contributions

A.J., A.G., M.D., M.R., and M.K. contributed to the study concept and design, conducted the literature review, and were involved in drafting and critically revising the manuscript for important intellectual content. A.J. and A.G. participated in drafting the revised manuscript, literature review, analysis, and interpretation of the revised version. M.D. contributed to proposal preparation, statistical analysis, and drafting the revised manuscript. A.G. and A.J. supervised the study, gathered data, and assisted with the literature review. All authors have read and approved the final version of the manuscript. Dr. Azadeh Goodarzi had full access to all of the data in this study and takes complete responsibility for the integrity of the data and the accuracy of the data analysis.

## Funding

The authors received no specific funding for this work.

## Ethics Statement

All collected data were kept confidential and analyzed without specific names attached. The study adhered to Helsinki ethical principles. The project was registered at Iran University of Medical Sciences with registration No. 1403‐3‐104‐31896, bearing the scientific title “Efficacy and Safety of Small Molecule Inhibitor Therapies for Vitiligo: A Systematic Review in Adults, Adolescents, and Children: A systematic review”. It was approved by the Research Council under the ethics code number IR.IUMS.FMD.REC.1403.320.

## Consent

The authors obtained consent to publish. The current manuscript contains no individual′s data. Therefore, consent to publish is not applicable.

## Conflicts of Interest

The authors declare no conflicts of interest.

## Transparency Statement

The lead author, Azadeh Goodarzi, affirms that this manuscript is an honest, accurate, and transparent account of the study being reported; no important aspects of the study have been omitted; and any discrepancies from the study as planned (and, if relevant, registered) have been explained.

## Data Availability

The data that support the findings of this study are available from the corresponding author upon reasonable request. The authors confirm that the data supporting the findings of this study are available within the article and its supplementary materials.
